# A Retrospective Case Series of Older Adults Requiring Electroconvulsive Therapy (ECT) in Surgical Theatres

**DOI:** 10.1192/bjo.2025.10518

**Published:** 2025-06-20

**Authors:** Jennifer Parker, Seona Duroux

**Affiliations:** Avon & Wiltshire Mental Health Partnership NHS Trust, Bristol, United Kingdom

## Abstract

**Aims:** This observational case series describes 11 patients who underwent emergency ECT under the care of the later life liaison psychiatry team at the Bristol Royal Infirmary over a 12-month period between February 2024 and January 2025. This represents a 5-fold increase in ECT delivery from previous years. We describe patients who required ECT treatment for their psychiatric illness but were deemed to be too medically unwell or too high risk for a general anaesthetic outside of surgical theatres in an acute hospital setting. The aim of this case series was to evaluate the volume of patients requiring emergency ECT and to understand the various clinical rationales for this.

ECT is an evidence-based intervention which is recommended for the treatment of severe depression, psychosis, catatonia and other conditions. Typically, ECT is delivered by psychiatrists in dedicated ECT suites located apart from acute hospitals – as is the case in Bristol. General anaesthesia is required to safely deliver ECT.

**Methods:** Case notes of the patients who underwent ECT in acute hospital surgical theatres were retrospectively reviewed and data was extracted on demographic features, medical and psychiatric history and details of ECT treatment. Clinical outcomes were measured using the Clinical Global Impressions (CGI) scale.

**Results:** 9 out of 11 patients who required emergency ECT in theatres were older adults (>65 years) with a skew towards advanced old age (>80). The most common reason for this treatment was severe depression and/or catatonia with associated need for enteral feeding due to not eating and drinking which required acute medical admission and increased anaesthetic risk. Other medical and surgical concerns included severe heart failure and uncontrolled Parkinson’s disease. Overall clinical improvement was seen across 9/11 of patients. 2 patients died within one month of undergoing ECT due to physical morbidity.

**Conclusion:** This case series which was conducted to evaluate a service being offered by the liaison psychiatry team illustrates the challenge of treating severely mentally unwell patients due to their associated poor physical health. We contend that ECT can (and sometimes should) be offered in the acute hospital when patients are too severely unwell or high risk to receive ECT in peripheral settings, which will particularly benefit those multimorbid older adults who stand to gain the most from ECT.

Given the volume of ECT being delivered and the apparent clinical necessity for this service, next steps will include additional training and service considerations including training liaison nurses in ECT skills.

